# Prescribing medications for attention-deficit/hyperactivity disorder in children: a retrospective analysis of a Japanese health insurance claims database

**DOI:** 10.1186/s12888-026-08147-1

**Published:** 2026-05-14

**Authors:** Ryutaro Suzuki, Ryosuke Kumazawa, Takumi Miyoshi, Junsei Tanaka, Taiyo Nishikawa, Hiroyuki Muraoka, Manabu Akazawa, Ken Inada

**Affiliations:** 1 Division of Integrated Psychosocial Care in Community and Child Psychiatry, Kitasato University School of Medicine, 1-15-1 Kitasato, Minami-ku, Sagamihara-shi, Kanagawa 252-0374 Japan; 2 Department of Psychiatry, Kitasato University School of Medicine, 1-15-1 Kitasato, Minami-ku, Sagamihara-shi, Kanagawa 252-0374 Japan; 3 Department of Public Health and Epidemiology, Meiji Pharmaceutical University, 2-522-1 Noshio, Kiyose-shi, Tokyo 204-8588 Japan; 4 The University of Tokyo Hospital, Office of Performance Monitoring and Risk Management, Bunkyo-ku, Tokyo 113-8655 Japan

**Keywords:** Attention deficit disorder with hyperactivity, Child, Adolescent, Drug prescriptions, Practice patterns, Japan

## Abstract

**Background:**

Despite pharmacotherapy being a significant treatment protocol for attention-deficit/hyperactivity disorder (ADHD), an increasingly common neurodevelopmental condition among children in Japan, prescribing practices for ADHD medications remain underexplored. This study examined prescribing patterns of medications for ADHD in children and adolescents aged 0–17 years in Japan and analyzed drug selection trends on the basis of patients’ backgrounds, including sex, age group, and medical department.

**Methods:**

In this retrospective database study using a large Japanese health insurance claims database, we analyzed prescription patterns from April 2018 to August 2023. We examined the proportion of patients prescribed each of the four ADHD medications, namely, Osmotic controlled-release oral delivery system methylphenidate (OROS-MPH), atomoxetine (ATX), guanfacine (GXR), and lisdexamfetamine (LDX), their combination patterns, and new prescription proportions across different demographic groups and medical departments. The Jonckheere–Terpstra trend test was employed to assess trends in prescription proportions over time. The Mann‒Whitney U test was employed to compare time series data on the proportion of monotherapy prescriptions in psychiatric and nonpsychiatric settings.

**Results:**

From 2018 to 2023, OROS-MPH prescriptions significantly decreased from 64.7% to 47.5%, whereas GXR prescriptions significantly increased from 19.3% to 46.4%. GXR was prescribed most frequently for female patients and children aged 6–12 years. For new patients, GXR prescriptions began exceeding those of OROS-MPH from late 2019 onward, comprising 41.3% of new prescriptions by 2023. The percentage of patients receiving monotherapy decreased from 82.3% to 79.6%, with OROS-MPH plus GXR being the most common combination therapy.

**Conclusions:**

In Japan, GXR has become an increasingly preferred medication for ADHD, particularly for female patients and children aged 6–12 years, with new prescriptions exceeding those of OROS-MPH since late 2019. This trend may be attributed to GXR not being subject to the same distribution control regulations as central nervous system stimulants and to its distinctive pharmacological mechanisms that differ from those of other ADHD medications. Continuous monitoring of prescription practices is necessary as treatment options continue to evolve.

**Clinical trial number:**

Not applicable

**Supplementary Information:**

The online version contains supplementary material available at 10.1186/s12888-026-08147-1.

## Background

Attention-deficit/hyperactivity disorder (ADHD) is a neurodevelopmental condition typically characterized by hyperactivity, impulsivity, and inattention, which often persist in adulthood and negatively affect schoolwork, employment, and relationships [1, 2]. The global prevalence of ADHD is estimated to be approximately 5.0% in children and adolescents [3], and epidemiological studies in Japan estimate the prevalence of ADHD in children to be 5.8% [4], with an increase in the estimated incidence [5]. However, prevalence varies considerably across countries, with some reporting rates as low as 1% [6]. Psychosocial treatments and pharmacotherapy tailored to an individual patient’s condition are recommended for ADHD [7]. Pharmacotherapy for ADHD is effective in children and adolescents [8] and is associated with a decreased risk of various functional outcomes associated with ADHD [9]. Pharmacotherapy is also recommended in treatment guidelines in Europe, the United States, and Japan [10–13]. Despite the effectiveness of pharmacotherapy and its recommendation in guidelines, many patients with ADHD remain untreated with central nervous system (CNS) stimulants, particularly in smaller countries. This situation may be influenced by various factors including regulatory restrictions and prescribing practices [14]. Over the past few decades, the prevalence of ADHD and the use of ADHD medications have increased in several countries [15–17], along with concerns about potential overdiagnosis and inappropriate prescription of ADHD medications [18, 19].

In the 2000s, the abuse of short-acting methylphenidate, which is used to treat conditions such as depression and pediatric ADHD in Japan (used off-label for the latter disorder), became a social problem [20]. Osmotic controlled-release oral delivery system methylphenidate (OROS-MPH) and atomoxetine (ATX) were subsequently administered to pediatric patients with ADHD between October 2007 and June 2009. Because of dependence and abuse, since 2008, distribution control regulations have been implemented for OROS-MPH, a CNS stimulant. Additionally, the fourth edition of the Japanese guidelines for the diagnosis and treatment of ADHD published in 2016 [21] lists the use of only OROS-MPH and ATX as medication options for ADHD. Guanfacine (GXR) and lisdexamfetamine (LDX) began being administered in June 2017 and December 2019, respectively. Currently, the registration of details of patients, prescribing physicians (who must be physicians with expertise), medical institutions, and dispensing pharmacies is required to prescribe the two CNS stimulants (OROS-MPH and LDX), and there is a 30-day limit on prescription. The latest guidelines (5th edition), published in 2022, list several ADHD medications, such as OROS-MPH, LDX, ATX, and GXR [12]. The guidelines list non-CNS stimulants ATX and GXR as first-line agents, along with OROS-MPH, and recommend their use as monotherapy. International evidence demonstrates that CNS stimulants have medium to large effect sizes and are generally more effective than non-CNS stimulants [22]. Accordingly, guidelines in the United States and Europe recommend CNS stimulants as first-line agents [11–13]. In contrast, Japan regulates the distribution of these CNS stimulants, consequently creating a unique treatment environment where non-CNS stimulants are positioned equally as first-line options. Since ADHD medications are intended for continuous use, the choice of therapeutic agent is critically important. The selection is based on patients’ individuality and physicians’ experiences with reference to guidelines, which are areas where further evidence needs to be accumulated. Knowing which drugs are most likely to be used for each patient will guide future drug selection. In addition, CNS stimulants carry the risks of dependence and abuse [23]. Therefore, to ensure safe drug use, prescription practices, such as changes in the therapeutic environment after a new drug is introduced, should be surveyed. In Japan, psychiatrists and pediatricians are mainly involved in the treatment of ADHD, and differences in the choice of therapeutic agents on the basis of physicians’ backgrounds are relatively unknown.

Understanding the unique Japanese treatment environment for ADHD requires accumulating evidence on actual prescribing practices. Previous studies in Japan have examined ADHD medication prescription patterns stratified by patient demographics and department type [24–27]. However, as these studies were based on data collected prior to LDX approval in December 2019, they could not examine the full therapeutic landscape with all four medications available. Additionally, comprehensive analyses of specific combination therapy patterns have remained limited. This study provides updated analyses using health insurance claims data from April 2018 to August 2023, including the period after all four medications became available. The study aims to clarify recent drug selection patterns based on patient backgrounds, examine trends in medications prescribed to newly initiating patients, and investigate detailed combination therapy patterns in current Japanese practice.

## Methods

### Data sources

Data were extracted from a large health insurance claims database developed and managed by the DeSC Healthcare Corporation (Tokyo, Japan). The DeSC database includes three types of insurance: national health insurance, employer-based health insurance, and the late-stage senior citizens’ healthcare system. All residents are included in Japan’s health insurance system. The type of public insurance does not affect medical care. Differences in public insurance are based on age, occupation, and employment status.

DeSC contains epidemiological claims data for approximately 12 million insurance enrollees, and the age and sex distributions of individuals listed in the database are similar to those in Japanese population estimates [28]. Since this study aimed to focus on early childhood and school-age children, it covers insurance claimants of national health insurance and employer-based health insurance. Children are covered by their parents’ insurance regardless of their medical condition. Therefore, they are consistently included in insurance claims data, minimizing the possibility of selection bias. Insurance claims include all inpatient, outpatient, and pharmacy claims received by the insurer, including those related to diagnoses, medical procedures, and prescribed drugs. Data were recorded even if the participants changed healthcare providers or used multiple providers. To our knowledge, this is the first study to use the DeSC database to examine ADHD medication prescribing patterns in a pediatric population.

### Patient selection

We adopted a retrospective database study design to understand prescribing practices while analyzing background factors. The dataset under consideration—the DeSC insurance claims database—was collected from April 1, 2014, to August 31, 2023. The analysis period was from April 1, 2018, to August 31, 2023. In this study, each period from April of one year to March of the following year was defined as one year. This period was further subdivided into four three-month periods. The temporal framework under consideration encompasses the following quarters: the initial quarter (Q1), from April to June; the second quarter (Q2), from July to September; the third quarter (Q3), from October to December; and the fourth quarter (Q4), from January to March of the following year. The initial month of this period was designated April in accordance with the Japanese school calendar.

The study population consisted of males and females aged 0–17 years who were diagnosed with ADHD and were registered in the DeSC database between April 1, 2014, and August 31, 2023 (ICD-10 code: F90). Furthermore, the participants were required to have received at least one dose of ADHD medication during the analysis period. The age of the participants was determined as the age on the first day of each period, that is, the first day of April, July, and October of one year, and January of the following year. The proportion of new prescriptions was determined by including patients for whom ADHD medications were initiated for the first time during the study period, as defined by the following criteria. Inclusion criteria for new prescription analysis: Patients were included in the new prescription analysis if they: (1) had no record of any ADHD medication prescription from the data access start date (April 1, 2014) to the index prescription date, (2) had at least 180 days of data enrollment before the index prescription, and (3) received their first ADHD medication prescription during the analysis period (April 1, 2018 to August 31, 2023).

Although the DeSC database includes diagnostic codes for all medical encounters, we did not extract or analyze data on psychiatric or medical comorbidities in this study. The focus was on overall prescribing patterns rather than examining the influence of specific comorbid conditions on medication selection. This limitation is discussed in the Limitations section.

### Outcomes

The outcome measures included the proportion of patients prescribed each ADHD medication relative to all patients prescribed any ADHD medication (prescription proportion), the proportion of each combination pattern of ADHD medications (prescription pattern proportion), the monotherapy proportion, and the proportion of patients newly initiating each ADHD medication (new prescription proportion). For each time period, we identified the number of unique patients who met the inclusion criteria and were prescribed ADHD medications. For prescription proportion calculations, each medication was evaluated independently. Patients receiving combination therapy were included in the count for each medication prescribed. For example, a patient receiving both OROS-MPH and GXR was counted in both the OROS-MPH prescription proportion and the GXR prescription proportion. Consequently, the sum of prescription proportions across all four medications may exceed 100%. These outcome measures were examined according to sex, age group (0–5, 6–12, and 13–17 years), and department (psychiatry/nonpsychiatry) at each time point.

### Statistical analysis

This retrospective database study included all patients who met the inclusion criteria, as this was a descriptive epidemiological study without hypothesis testing. The medications used to treat ADHD included various formulations of the following four drugs: OROS-MPH, LDX, ATX, and GXR. There was no minimum requirement for the number of prescriptions; patients prescribed medication only once were included if they met the criteria. The proportion of prescriptions was calculated in the following manner. The duration of drug therapy was calculated from the start date and the number of days on which each drug was prescribed. The drug was considered to have been prescribed to patients during the duration of the drug. For each of the four medications (OROS-MPH, LDX, ATX, and GXR), we identified all patients who were prescribed that medication during each period. The prescription proportion for each medication was calculated using the number of patients prescribed that medication as the numerator and the total number of patients prescribed any ADHD medication as the denominator. Patients receiving combination therapy were counted in the numerator for each medication prescribed; thus, the same patient could be counted in multiple medication groups simultaneously. The proportion of new prescriptions was calculated similarly.

The proportion of the prescription pattern was calculated in the following manner. Fifteen distinct combinations of concomitant prescriptions, contingent on the prescription of each of the four drug groups, existed (monotherapy: four patterns; two-drug combination: six patterns; three-drug combination: three patterns; four-drug combination: one pattern). To calculate the proportion of prescription patterns in each period, the number of patients per prescription pattern was designated the numerator, and the total number of patients for whom ADHD medications were prescribed was designated the denominator. A minimum of three distinct pharmacological agents were aggregated into a single category. The monotherapy proportion was calculated using the total of four monotherapy patterns as the numerator and the total number of patients prescribed ADHD medications as the denominator.

Psychiatric versus nonpsychiatric care was distinguished on the basis of billing codes indicating psychotherapy services, which are generally calculated via insurance coverage in psychiatric institutions in Japan.

The Jonckheere–Terpstra trend test was employed to assess trends in prescription proportions over time. The Mann‒Whitney U test was employed to compare time series data on the proportion of monotherapy prescriptions in psychiatric and nonpsychiatric settings. The significance level was set at 5%. Bonferroni correction was used to address the issue of multiplicity. Microsoft SQL Server (version 2022) was used for database processing, whereas SAS (version 9.4) and RStudio (version 4.4.0) were used for data analysis.

### Ethics

The Research Ethics Review Committee of Meiji Pharmaceutical University approved this study on June 27, 2024 (registration number: 202408). As the data used in this study were unlinked and anonymous, informed consent was not needed.

## Results

### Trends in prescription proportions

**Fig. 1:**
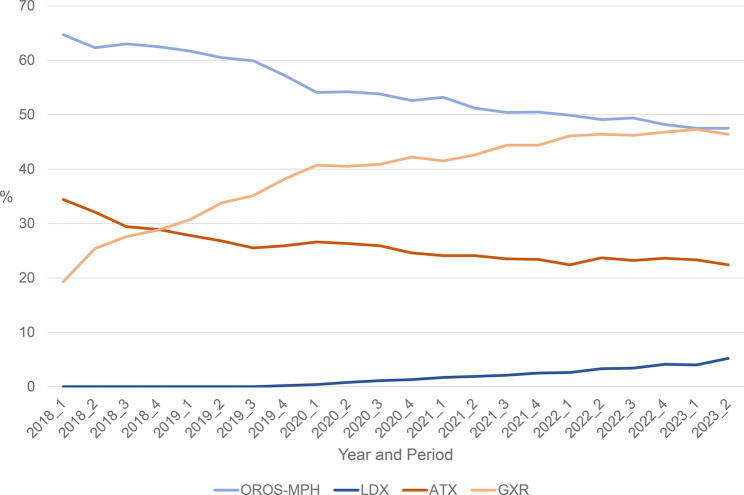
Overall proportion of prescriptions of ADHD medications. OROS-MPH: Osmotic controlled-release oral delivery system methylphenidate, LDX: Lisdexamfetamine, ATX: Atomoxetine, GXR: Guanfacine

**Fig. 2:**
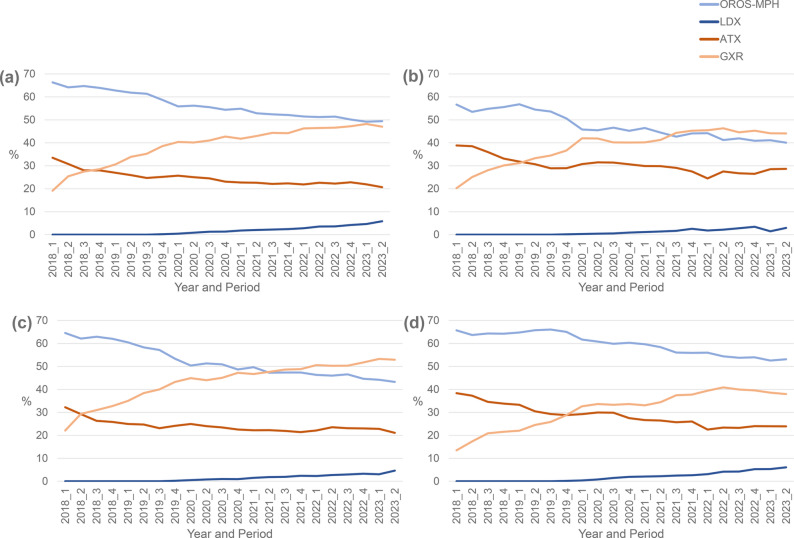
**a** overall prescription proportions for boys. **b** overall prescription proportions for girls. **c** overall prescription proportions for 6–12-year-olds. **d** overall prescription proportions for 13–17-year-olds. OROS-MPH: Osmotic controlled-release oral delivery system methylphenidate, LDX: Lisdexamfetamine, ATX: Atomoxetine, GXR: Guanfacine

A total of 72,609 patient observations were analyzed across 22 quarterly periods (Q1/2018 to Q2/2023). Table 1 shows the patient profiles. Boys outnumbered girls, and the 6–12 age group comprised the highest number of patients. Detailed trends in prescription are shown with numerical data in Appendix Table S1. The proportion of prescriptions for each ADHD medication is outlined in Fig. 1, with sex- and age-specific trends shown in Fig. 2a–d. A significant downward trend of 64.7% to 47.5% (*p* < 0.001) was observed for OROS-MPH from Q1/2018 to Q2/2023; a significant downward trend (*p* < 0.001) of 34.4% to 22.4% for ATX; and a significant upward trend (*p* < 0.001) of 19.3% to 46.4% for GXR. GXR surpassed ATX in Q1/2019 and, more recently, surpassed OROS-MPH in prescription proportion. LDX showed a significant increasing trend (*p* < 0.001) from Q4/2019 (market launch) to Q2/2023, reaching 5.2%. In terms of sex (Fig. 2a and b), GXR was greater than OROS-MPH in girls at Q3/2021. By age group (Fig. 2c and d), GXR exceeded OROS-MPH in the middle of the observation period in the 6–12 age group, but OROS-MPH consistently exceeded GXR in the 13–17 age group. By department (Table 2), OROS-MPH was the most prescribed drug in nonpsychiatric departments, whereas in psychiatric departments, GXR surpassed OROS-MPH in the middle of the observation period and remained higher in the most recent period.

**Table 1:** Characteristics of the prescribed patients

Year		2018				2019				2020				2021				2022			2023	
	1	2	3	4	1	2	3	4	1	2	3	4	1	2	3	4	1	2	3	4	1	2
*Total*	2874	3013	3128	3096	3304	3448	3525	3457	3524	3737	3889	3893	3849	3927	4069	4014	3328	3323	3313	3059	1542	1297
*Boys*	2385	2499	2597	2565	2723	2826	2878	2835	2893	3042	3138	3133	3083	3147	3237	3193	2618	2600	2595	2421	1209	1025
*0–5 years*	10	19	24	21	16	17	18	12	9	21	24	23	17	18	24	16	12	16	9	8	4	1
*6–12 years*	1626	1702	1740	1700	1839	1916	1925	1888	1941	2024	2047	2010	1951	2004	2049	1993	1586	1583	1578	1467	732	594
*13–17 years*	749	778	833	844	868	893	935	935	943	997	1067	1100	1115	1125	1164	1184	1020	1001	1008	946	473	430
*Girls*	489	514	531	531	581	622	647	622	631	695	751	760	766	780	832	821	710	723	718	638	333	272
*0–5 years*	3	3	4	3	1	2	4	1	5	9	7	5	5	5	5	4	6	8	2	4	0	0
*6–12 years*	307	320	325	314	349	388	393	362	365	407	430	439	428	434	467	464	396	402	400	359	184	141
*13–17 years*	179	191	202	214	231	232	250	259	261	279	314	316	333	341	360	353	308	313	316	275	149	131

^1^Periods represent three-month quarters: Period 1 (April–June), Period 2 (July–September), Period 3 (October–December), and Period 4 (January–March of the following year), following the Japanese fiscal year

**Table 2:** Comparison of psychiatric and nonpsychiatric prescriptions for ADHD medications

Year		2018				2019				2020				2021				2022			2023	
Period^1^	1	2	3	4	1	2	3	4	1	2	3	4	1	2	3	4	1	2	3	4	1	2
**Psychiatry**																						
*Total*	139	146	159	170	190	213	217	205	195	206	204	211	203	218	220	205	155	171	175	170	58	49
*OROS-MPH*^*2*^	89	88	99	91	103	115	112	95	81	86	87	90	95	96	103	97	73	72	75	67	18	18
*LDX*^*3*^	0	0	0	0	0	0	0	3	5	5	6	5	6	6	6	4	4	8	10	13	3	3
*ATX*^*4*^	46	41	36	35	45	54	53	47	46	43	47	46	51	45	46	51	48	54	46	46	19	16
*GXR*^*5*^	26	40	54	69	76	93	96	99	100	100	90	105	92	105	103	93	64	79	74	74	28	22
**Nonpsychiatry**																						
*Total*	2753	2892	2995	2951	3139	3264	3341	3283	3369	3573	3718	3712	3676	3759	3883	3850	3199	3191	3172	2913	1492	1253
*OROS-MPH*^*2*^	1781	1802	1887	1853	1949	1983	2015	1893	1831	1955	2018	1971	1963	1937	1962	1946	1602	1572	1573	1416	717	599
*LDX*^*3*^	0	0	0	0	0	0	0	4	11	26	40	45	62	71	83	98	82	102	107	113	59	65
*ATX*^*4*^	945	936	888	867	879	874	852	851	901	947	966	918	887	910	918	897	707	743	729	680	343	277
*GXR*^*5*^	535	730	817	834	947	1085	1157	1246	1356	1430	1515	1561	1520	1599	1720	1712	1482	1487	1475	1371	708	581

^1^Periods represent three-month quarters: Period 1 (April–June), Period 2 (July–September), Period 3 (October–December), and Period 4 (January–March of the following year), following the Japanese fiscal year

^2^OROS-MPH: Osmotic controlled-release oral delivery system methylphenidate, ^3^ LDX: Lisdexamfetamine, ^4^ ATX: Atomoxetine, ^5^ GXR: Guanfacine

### Trends in new prescriptions

**Fig. 3:**
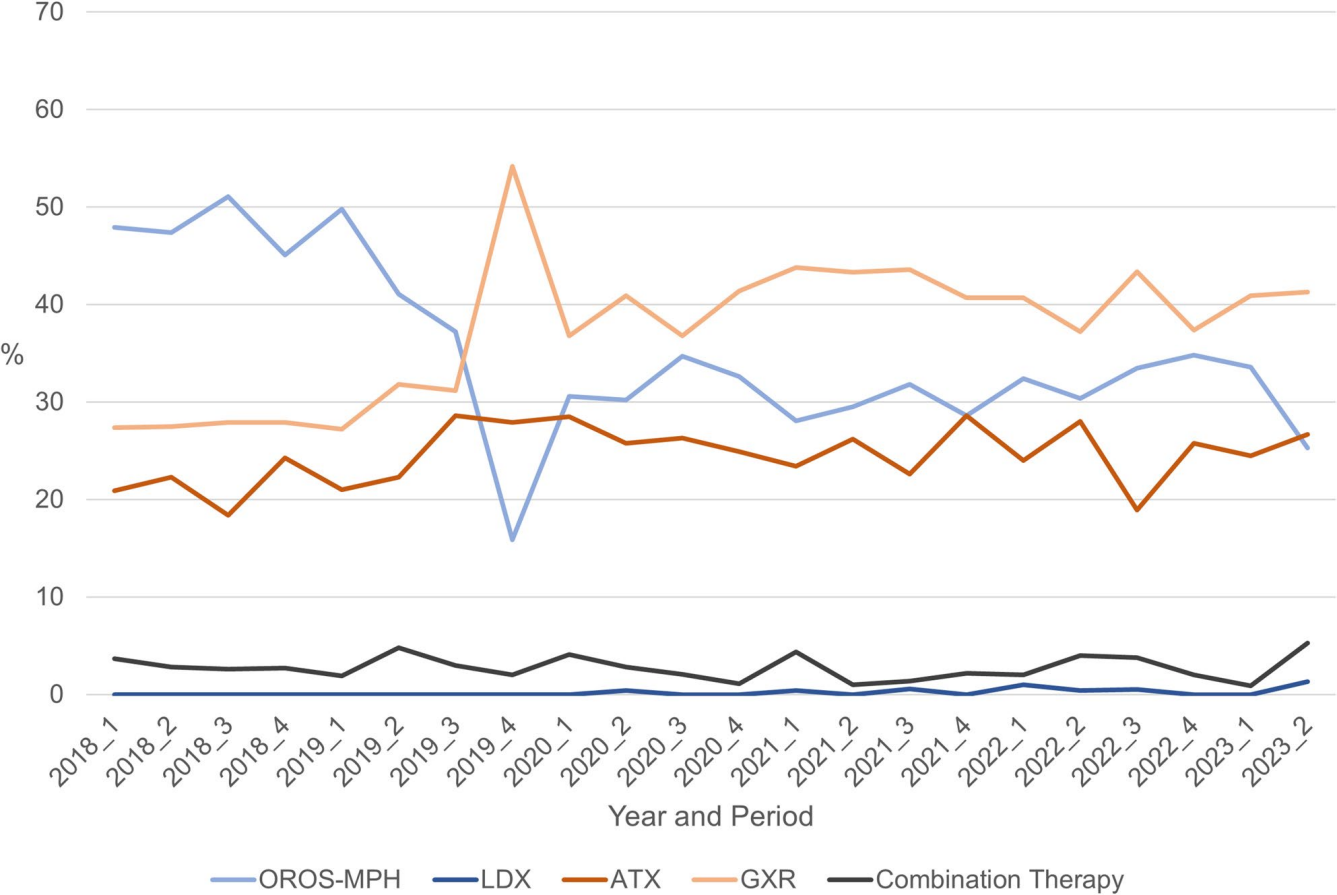
New prescription proportions of ADHD medications. OROS-MPH: Osmotic controlled-release oral. delivery system methylphenidate, LDX: Lisdexamfetamine, ATX: Atomoxetine, GXR: Guanfacine

Table 3 shows the profiles of patients receiving new prescriptions. Detailed trends in new prescription are shown with numerical data in Appendix Table S2. The proportion of new prescriptions for each ADHD medication (Fig. 3) significantly decreased from 47.9% to 25.3% for OROS-MPH from Q1/2018 to Q2/2023 (*p* = 0.0083). In contrast, an increasing trend of 20.9% to 26.7% (*p* = 0.16) and a significantly increasing trend of 27.4% to 41.3% (*p* = 0.0021) were observed for ATX and GXR, respectively. LDX changed from 0% to 1.3% (*p* = 0.030), and ADHD medication combinations changed from 3.7% to 5.3% (*p* = 0.45). Since Q4/2019, GXR has surpassed OROS-MPH and has remained at approximately 40%. In particular, in Q4/2019, the increase in GXR and decrease in OROS-MPH were more noticeable than they were in the periods before and after it.

**Table 3:** Characteristics of new prescription patients

Year		2018				2019				2020				2021				2022			2023	
Period^1^	1	2	3	4	1	2	3	4	1	2	3	4	1	2	3	4	1	2	3	4	1	2
*Total*	215	247	272	226	261	291	267	202	199	252	279	284	274	298	349	273	204	250	212	198	110	75
*Boys*	161	191	212	171	205	217	203	154	140	185	195	202	199	218	251	194	139	174	148	140	78	54
*0–5 years*	1	5	5	2	0	4	3	1	2	8	8	2	1	4	3	1	1	2	0	0	1	1
*6–12 years*	125	139	160	111	163	170	152	119	103	141	148	151	154	181	201	139	97	134	117	107	57	35
*13–17 years*	35	47	47	58	38	44	50	33	29	36	45	50	44	33	47	54	41	38	31	33	20	18
*Girls*	54	56	60	55	56	74	64	48	59	67	84	82	75	80	98	79	65	76	64	58	32	21
*0–5 years*	1	0	1	0	0	1	0	0	2	3	1	1	1	1	0	0	1	0	0	1	0	0
*6–12 years*	34	36	37	30	30	47	37	21	28	38	52	38	42	41	63	42	37	43	36	32	18	10
*13–17 years*	19	20	22	25	26	26	27	27	29	26	31	43	32	38	35	37	27	33	28	25	14	11

^1^Periods represent three-month quarters: Period 1 (April–June), Period 2 (July–September), Period 3 (October–December), and Period 4 (January–March of the following year), following the Japanese fiscal year

### Trends in prescription pattern proportions

**Fig. 4:**
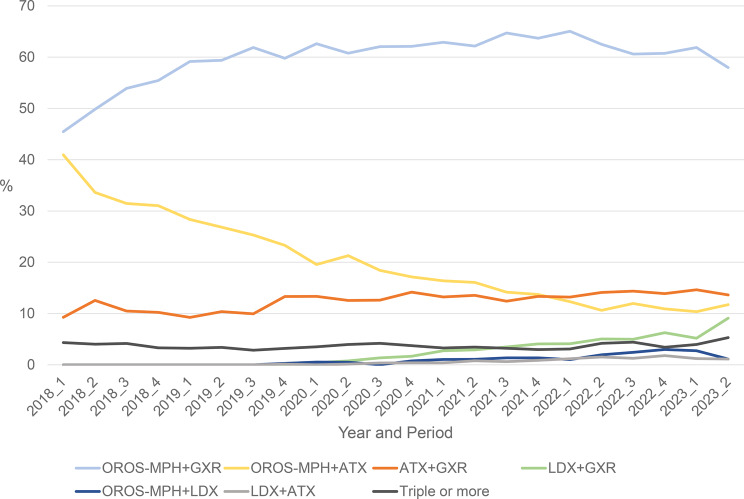
Trends in combination therapy patterns of ADHD medications. OROS-MPH: Osmotic controlled-release oral delivery system methylphenidate, LDX: Lisdexamfetamine, ATX: Atomoxetine, GXR: Guanfacine

The prescription pattern proportions are shown in Fig. 4, with detailed numerical data provided in Appendix Table S3. The overall monotherapy proportion for ADHD medications decreased significantly from 82.3% in Q1/2018 to 79.6% in Q2/2023 (*p* = 0.0011). When analyzed by department, the monotherapy proportion among psychiatric departments changed from 85.6% to 79.6%, but the trend was not consistent (*p* = 0.054), whereas the monotherapy proportion among nonpsychiatric departments significantly decreased from 82.2% to 79.7% (*p* < 0.001). A statistically significant difference between psychiatry and nonpsychiatry regarding the proportion of patients receiving monotherapy (*p* < 0.001) was also observed. When restricted to prescriptions for two or more drugs (Fig. 4), OROS-MPH + GXR showed a significant increasing trend from 45.5% to 58.0% between Q1/2018 and Q2/2023 (*p* = 0.0017). For medications administered concomitantly with LDX, the LDX + ATX proportion was 1.1% in Q2/2023, and the LDX + GXR proportion was 9.1% in Q2/2023. For the concomitant use of three or more drugs, the proportion changed from 4.3% to 5.3% from Q1/2018 to Q2/2023 (*p* = 0.82).

## Discussion

### Prescription proportions

To our knowledge, this study is the first to investigate prescription patterns of four ADHD medications since they became available in Japan. GXR prescriptions are increasing for both sexes and all ages, representing the overall trend in ADHD medication use. Distribution control regulations suppress CNS stimulant prescriptions [29]. Since GXR is not subject to these regulations, it is prescribed more readily than CNS stimulants because more physicians and medical institutions can prescribe it.

Japan’s regulatory approach to CNS stimulants and its impact on prescribing patterns contrast sharply with international practices. Most international guidelines, including those from the United States [13], Europe [11], and Canada [10], explicitly recommend CNS stimulants as preferred first-line agents based on their medium to large effect sizes and robust evidence base [16, 22]. While some countries, such as Spain, position CNS stimulants and non-CNS stimulants as parallel treatment options in clinical guidelines [30], methylphenidate remains the predominant choice, accounting for 91% of patients in Spain [31]. Similarly, in Asian countries where both methylphenidate and atomoxetine are listed as treatment options, methylphenidate is typically the primary medication in clinical practice [32]. Japan is unique in both guideline positioning and actual prescribing patterns, where non-CNS stimulant use (particularly GXR at 46.4%) has reached levels equivalent to or exceeding CNS stimulant use (OROS-MPH at 47.5% in Q2/2023). Japan’s high non-CNS stimulant prescription rate appears primarily driven by strict regulatory constraints on CNS stimulant distribution. In Slovenia, the introduction of new medications and intensified pharmaceutical marketing led to decreased prescription of guideline-recommended first-line medications [33]. Investigating actual prescribing practices when new medications become available is important to monitor potentially inappropriate prescribing patterns.

During the COVID-19 pandemic, ADHD medication consumption increased in Europe, the United States, and Japan; however, in Europe and the United States, the share of CNS stimulants did not decrease [34, 35]. The United States and Europe eased restrictions on prescribing CNS stimulants. In Japan, regional states of emergency were declared intermittently (April–May 2020, January–March 2021, April–June 2021, and July–September 2021). During these periods, telehealth allowed only continued OROS-MPH prescriptions for patients already receiving treatment; new OROS-MPH prescriptions and all LDX prescriptions were not permitted via telehealth. This more restrictive telemedicine policy for CNS stimulants may have influenced the increase in non-CNS stimulants in Japan.

In recent years, awareness of ADHD has increased, and patients diagnosed with ADHD now include individuals with less severe presentations [36]. Non-CNS stimulants may be preferred for patients who can be treated without CNS stimulants. Among non-CNS stimulants, GXR has demonstrated superior efficacy compared with ATX in clinical trials [37–39], and unlike ATX, GXR maintains effectiveness even in patients with prior CNS stimulant exposure [40]. Therefore, GXR is more likely prescribed for patients after CNS stimulant use. Nonadherence to psychotropic medication is influenced by missed doses [41], and patients taking fewer daily doses are more likely to adhere to treatment [42]. ADHD’s developmental characteristics, such as inattentive tendencies, may also contribute to nonadherence [43]. From a dosage perspective, GXR, a once-daily medication, may be more likely selected than ATX, a twice-daily medication.

In girls, GXR prescriptions exceeded OROS-MPH prescriptions. Females are less severely affected by ADHD characteristics than males, suggesting that a larger proportion of patients can be treated with non-CNS stimulants. Female patients are often diagnosed primarily with inattentive ADHD and have fewer externalizing problem behaviors, which may contribute to missed diagnoses [44] or lower likelihood of initiating pharmacotherapy unless they have significant externalizing problems [45].

The highest proportion of GXR prescriptions was for children aged 6–12 years. Compared with placebo, GXR improved the Conners’ subscale score for defiant challenge and total ADHD Rating Scale scores in children with ADHD aged 6–12 with defiant challenge tendencies [46]. GXR may be used for children who are not physically large or for preadolescents. Most studies on GXR efficacy for pediatric ADHD have focused on children aged 6–17 years, and further evidence is needed regarding ADHD medication efficacy in subdivided age groups.

Child psychiatrists had significantly more follow-up visits after initiating pharmacotherapy than primary care physicians [47]. In Japan’s outpatient insurance system, child psychiatrists tend to have longer consultation times than nonpsychiatrists. Psychiatric consultations have a minimum requirement of at least 5 minutes for insurance reimbursement purposes, whereas in nonpsychiatric settings (mainly pediatrics), children with ADHD are often seen alongside patients presenting with physical symptoms such as colds and fever, resulting in a higher number of patients seen per hour compared to psychiatric settings. Nonpsychiatric departments face greater difficulty in allocating sufficient time to individual patients, primarily due to economic constraints. Psychiatrists, with more frequent follow-up opportunities and longer consultation times, can provide more intensive nonpharmacological interventions, potentially leading to a higher proportion of patients being treated with non-CNS stimulants. While differences in consultation practices between specialties have been observed, the overall capacity of the healthcare system to provide ADHD care also warrants consideration. As of December 2022, there were 17,781 pediatricians (5.4% of all physicians) and 16,817 psychiatrists (5.1%) in Japan [48]. However, not all pediatricians provide ADHD care, and some psychiatrists do not treat children; therefore, the actual number of physicians involved in ADHD care remains unknown. The observed differences in prescribing patterns between psychiatric and nonpsychiatric settings may reflect broader systemic factors in ADHD care delivery. While the present study focused on prescription patterns rather than healthcare system structures, these findings suggest that care delivery models warrant further consideration. International approaches, such as the United Kingdom’s shared care arrangement where general practitioners conduct routine follow-up after specialist-led diagnosis and treatment initiation [49], represent potential frameworks for consideration, though their applicability and effectiveness in the Japanese healthcare context require further investigation.

### New prescription proportions

GXR has become increasingly preferred among new prescriptions. The proportion of LDX in new prescriptions was 1.3%, and concomitant use was 5.3%. Time series data show that new prescriptions generally started with OROS-MPH, ATX, or GXR as monotherapy, consistent with guideline recommendations. A temporary change was observed in time series data for Q4/2019. This period coincided with the COVID-19 pandemic onset, during which outpatient visits to pediatric clinics decreased [50]. Uncertainty regarding healthcare system outlook may have influenced OROS-MPH avoidance, which has a limited prescription duration.

### Prescription pattern proportions

In recent years, GXR was more likely chosen than ATX in combination with OROS-MPH. The overall trend for ADHD medications is the greater likelihood of selecting GXR, which influences the increasing proportion of prescription patterns involving GXR in combination therapy. Previous studies on combination therapy, including literature reviews [51], have compared OROS-MPH and ATX with placebo [52], OROS-MPH and GXR with placebo [53], and GXR with CNS stimulants, including OROS-MPH and LDX, with placebo [51, 54]. However, no studies have directly compared combination therapies. Pharmacologically, OROS-MPH inhibits dopamine and noradrenergic transporters, whereas ATX inhibits the noradrenergic transporter; both share the same noradrenergic transporter inhibitory effect. Conversely, GXR’s pharmacological action is the selective activation of α2A receptors, a mechanism different from OROS-MPH and ATX. GXR may be more likely an adjunctive agent for OROS-MPH when monotherapy is inadequate and more likely used in combination with drugs with different pharmacological actions. Further studies are warranted to establish optimal combination therapy use.

The increasing use of combination therapy raises important considerations for patient management. Given the potential for cumulative or overlapping adverse effects from multiple medications, clinicians should carefully monitor adverse events in patients receiving combination therapy. It is important to establish systematic approaches to evaluate incremental benefit and tolerability when adding a second medication, rather than assuming combination therapy is uniformly superior to optimized monotherapy. Clinicians should avoid continuing combination therapy without clear clinical rationale and should periodically reassess whether both medications remain necessary for optimal symptom control.

Increased drug therapy options led to increased combination therapy options, and the overall monotherapy proportion declined. Significant differences were observed in monotherapy proportions between psychiatry and nonpsychiatry departments, with no trend in increase or decrease among psychiatry departments. Monotherapy is recommended in psychiatry for other psychiatric disorders, such as schizophrenia and depression, which may influence the monotherapy proportion among psychiatrists aware of its benefits. Compared with monotherapy proportions for other psychiatric disorders of 57.1% for schizophrenia and 58.6% for depression [55, 56], the approximately 80% monotherapy proportion for ADHD medications is high.

## Limitations

This study has several limitations that should be considered when interpreting the results. First, administrative claims data used in this retrospective database study have inherent limitations. Claims data primarily capture billed services and dispensed medications, and may not fully reflect clinical decision-making processes, treatment rationale, or patient-specific factors that influence medication selection. The database does not include information about the clinical context of prescribing decisions, such as symptom severity, treatment response, or reasons for medication changes. Furthermore, we analyzed prescription patterns at the population level and cannot determine outcomes at the individual patient level. Second, several important data limitations affect this study. The severity of ADHD was not considered in this study because it was not included in the database. The timing of data provision by insurance associations to DeSC varies; in some instances, the preceding year’s data were enrolled as lump sums at the conclusion of the subsequent year. As the data presented herein were collected in January 2024, a decline in the number of enrolled patients has been observed since 2022. Consequently, the number of eligible patients, particularly for new prescription and combination therapy analyses, was minimal, which impedes our capacity to conduct extensive analyses stratified by sex, age, and department. Subsequent analyses will require accumulation of sufficient patient numbers in the database, and use of alternative databases should also be considered. Additionally, in the selection of new prescribing patients, the first prescriptions for patients who had been registered in the database for at least six months were identified; prescriptions prior to March 31, 2014, were not registered in the database. Consequently, individuals who were taking medications prior to March 31, 2014, and resumed taking them after April 1, 2018, may have been incorrectly classified as newly prescribed patients. Furthermore, if a patient transferred from an alternative insurance association and subsequently registered in the database, a patient who resumed medication after a withdrawal period of six months or longer would be treated as a newly prescribed patient, because the medication status of patients more than six months prior is unknown. Third, the classification of psychiatric versus nonpsychiatric care was based on the presence of billing codes for psychotherapy services on the same insurance claim as the ADHD medication prescription. Because this definition relies on claim-level co-occurrence rather than the actual clinical setting, the number of patients classified as receiving psychiatric care may not accurately reflect the true number of patients treated by psychiatrists. Fourth, ADHD frequently coexists with various psychiatric and neurodevelopmental disorders, such as autism spectrum disorder, anxiety disorders, mood disorders, and learning disabilities. These comorbidities can significantly influence medication selection, treatment response, and overall treatment strategies. We did not examine comorbid diagnoses or concomitant use of other psychotropic medications (e.g., antidepressants, antipsychotics, anxiolytics), which may confound the observed prescription patterns. Fifth, this study examined prescription patterns but could not assess actual medication adherence or treatment persistence. In claims databases, a prescription does not guarantee that the patient actually took the medication as prescribed. The dispensing of a medication indicates only that a prescription was filled, not that the medication was consumed or taken according to instructions. Non-adherence to ADHD medications is common and may vary across different medications, patient ages, and clinical contexts. Without adherence measures and clinical outcomes data, it is difficult to fully evaluate the real-world clinical implications of these prescribing patterns. Future research combining prescription data with adherence measures and treatment outcomes would provide valuable insights into the clinical significance of these prescribing trends. Sixth, in the context of combination therapy, the analytical approach employed in this study involves categorizing each medication modification as combination therapy, irrespective of alterations in individual medications implemented over a three-month period. Consequently, the prescription proportion for combination therapy may exceed the actual proportion of patients receiving truly concurrent combination therapy, whereas the proportion for monotherapy may fall below the actual proportion. This methodological approach means that patients who switched from one medication to another within a three-month period would be counted as receiving combination therapy, even if the medications were not used simultaneously.

## Conclusion

In Japan, a trend toward a growing proportion of GXR prescriptions for ADHD has been observed. In particular, GXR has been identified as the most frequently prescribed medication among female patients and pediatric patients aged 6–12 years. GXR has become the preferred first-line medication for new patients and is increasingly chosen for combination therapy regimens. This trend may be attributed to GXR not being a CNS stimulant; consequently, it is not subject to the same distribution control regulations as OROS-MPH and LDX. Furthermore, the different pharmacological effects of GXR compared with those of OROS-MPH and ATX may also contribute to this trend. Future studies should evaluate the long-term clinical effectiveness and safety of these shifting prescription patterns, particularly the increased use of GXR, to optimize ADHD treatment strategies for Japanese children.

## Electronic supplementary material

Below is the link to the electronic supplementary material.

Supplementary material 1

## Data Availability

The data supporting the findings of this study were obtained from DeSC Healthcare Corporation (Tokyo). The use of these data is subject to restrictions and was licensed for use in this study. Therefore, supporting data are not available.

## Abbreviations

ADHD: Attention-deficit/hyperactivity disorder
OROS-MPH: Osmotic controlled-release oral delivery system methylphenidate
ATX: Atomoxetine
GXR: Guanfacine
LDX: Lisdexamfetamine
CNS: Central nervous system
