# Supplementary material for: Prescribing medications for attention-deficit/hyperactivity disorder in children: a retrospective analysis of a Japanese health insurance claims database
Source: BMC Psychiatry. 2026 May 14;26:525. doi: 10.1186/s12888-026-08147-1 (PMC13348584; doi:10.1186/s12888-026-08147-1)
Supplement: Supplementary file 1 — Supplementary material 1 [file 12888_2026_8147_MOESM1_ESM.docx]

**Table S1.** *Trends in the prescription of ADHD medications*

| *Year* |  | 2018 | |  |  | 2019 | |  |  | 2020 | |  |  | 2021 | |  |  | 2022 | |  | 2023 | |
| --- | --- | --- | --- | --- | --- | --- | --- | --- | --- | --- | --- | --- | --- | --- | --- | --- | --- | --- | --- | --- | --- | --- |
| *Period¹* | 1 | 2 | 3 | 4 | 1 | 2 | 3 | 4 | 1 | 2 | 3 | 4 | 1 | 2 | 3 | 4 | 1 | 2 | 3 | 4 | 1 | 2 |
| **OROS-MPH²** |  |  |  |  |  |  |  |  |  |  |  |  |  |  |  |  |  |  |  |  |  |  |
| *Total* | 1859 | 1878 | 1972 | 1935 | 2040 | 2087 | 2113 | 1979 | 1905 | 2026 | 2092 | 2049 | 2047 | 2011 | 2051 | 2026 | 1662 | 1630 | 1635 | 1475 | 732 | 616 |
| *Boys* | 1582 | 1603 | 1681 | 1640 | 1710 | 1748 | 1766 | 1664 | 1616 | 1710 | 1742 | 1705 | 1691 | 1664 | 1696 | 1664 | 1348 | 1332 | 1334 | 1214 | 595 | 507 |
| *0–5 years* | 1 | 5 | 6 | 5 | 5 | 3 | 5 | 1 | 0 | 2 | 4 | 3 | 1 | 1 | 5 | 3 | 0 | 1 | 2 | 1 | 0 | 0 |
| *6–12 years* | 1076 | 1091 | 1129 | 1085 | 1137 | 1157 | 1142 | 1045 | 1018 | 1081 | 1073 | 1006 | 994 | 976 | 1005 | 976 | 754 | 763 | 770 | 685 | 338 | 269 |
| *13–17 years* | 505 | 507 | 546 | 550 | 568 | 588 | 619 | 618 | 598 | 627 | 665 | 696 | 696 | 687 | 686 | 685 | 594 | 568 | 562 | 528 | 257 | 238 |
| *Girls* | 277 | 275 | 291 | 295 | 330 | 339 | 347 | 315 | 289 | 316 | 350 | 344 | 356 | 347 | 355 | 362 | 314 | 298 | 301 | 261 | 137 | 109 |
| *0–5 years* | 0 | 0 | 0 | 0 | 0 | 0 | 0 | 0 | 0 | 0 | 0 | 0 | 0 | 1 | 0 | 0 | 0 | 1 | 0 | 0 | 0 | 0 |
| *6–12 years* | 172 | 165 | 171 | 165 | 186 | 187 | 183 | 156 | 144 | 167 | 188 | 186 | 188 | 177 | 187 | 188 | 164 | 150 | 151 | 130 | 67 | 49 |
| *13–17 years* | 105 | 110 | 120 | 130 | 144 | 152 | 164 | 159 | 145 | 149 | 162 | 158 | 168 | 169 | 168 | 174 | 150 | 147 | 150 | 131 | 70 | 60 |
| **LDX³** |  |  |  |  |  |  |  |  |  |  |  |  |  |  |  |  |  |  |  |  |  |  |
| *Total* | 0 | 0 | 0 | 0 | 0 | 0 | 0 | 7 | 15 | 29 | 44 | 49 | 65 | 76 | 86 | 99 | 86 | 109 | 114 | 124 | 61 | 68 |
| *Boys* | 0 | 0 | 0 | 0 | 0 | 0 | 0 | 6 | 13 | 26 | 40 | 42 | 56 | 65 | 72 | 78 | 73 | 93 | 94 | 102 | 56 | 60 |
| *0–5 years* | 0 | 0 | 0 | 0 | 0 | 0 | 0 | 0 | 0 | 0 | 0 | 0 | 0 | 0 | 0 | 1 | 0 | 0 | 0 | 0 | 0 | 0 |
| *6–12 years* | 0 | 0 | 0 | 0 | 0 | 0 | 0 | 4 | 10 | 18 | 23 | 20 | 31 | 40 | 44 | 47 | 38 | 48 | 47 | 47 | 24 | 28 |
| *13–17 years* | 0 | 0 | 0 | 0 | 0 | 0 | 0 | 2 | 3 | 8 | 17 | 22 | 25 | 25 | 28 | 30 | 35 | 45 | 47 | 55 | 32 | 32 |
| *Girls* | 0 | 0 | 0 | 0 | 0 | 0 | 0 | 1 | 2 | 3 | 4 | 7 | 9 | 11 | 14 | 21 | 13 | 16 | 20 | 22 | 5 | 8 |
| *0–5 years* | 0 | 0 | 0 | 0 | 0 | 0 | 0 | 0 | 0 | 0 | 0 | 0 | 0 | 0 | 0 | 0 | 0 | 0 | 0 | 0 | 0 | 0 |
| *6–12 years* | 0 | 0 | 0 | 0 | 0 | 0 | 0 | 1 | 1 | 1 | 1 | 2 | 4 | 4 | 4 | 11 | 7 | 6 | 11 | 13 | 4 | 6 |
| *13–17 years* | 0 | 0 | 0 | 0 | 0 | 0 | 0 | 0 | 1 | 2 | 3 | 5 | 5 | 7 | 10 | 10 | 6 | 10 | 9 | 9 | 1 | 2 |
| **ATX⁴** |  |  |  |  |  |  |  |  |  |  |  |  |  |  |  |  |  |  |  |  |  |  |
| *Total* | 989 | 968 | 919 | 895 | 919 | 924 | 898 | 894 | 938 | 981 | 1006 | 957 | 929 | 945 | 958 | 940 | 747 | 788 | 770 | 721 | 360 | 290 |
| *Boys* | 799 | 770 | 728 | 719 | 734 | 733 | 711 | 714 | 744 | 762 | 770 | 724 | 700 | 712 | 716 | 714 | 573 | 589 | 578 | 552 | 265 | 212 |
| *0–5 years* | 8 | 13 | 15 | 14 | 6 | 10 | 11 | 6 | 8 | 10 | 11 | 13 | 13 | 12 | 13 | 12 | 6 | 9 | 4 | 5 | 2 | 1 |
| *6–12 years* | 514 | 483 | 443 | 431 | 450 | 457 | 428 | 447 | 477 | 470 | 465 | 438 | 411 | 421 | 420 | 404 | 346 | 366 | 356 | 338 | 167 | 124 |
| *13–17 years* | 277 | 274 | 270 | 274 | 278 | 266 | 272 | 261 | 259 | 282 | 294 | 273 | 276 | 279 | 283 | 298 | 221 | 214 | 218 | 209 | 96 | 87 |
| *Girls* | 190 | 198 | 191 | 176 | 185 | 191 | 187 | 180 | 194 | 219 | 236 | 233 | 229 | 233 | 242 | 226 | 174 | 199 | 192 | 169 | 95 | 78 |
| *0–5 years* | 2 | 3 | 3 | 3 | 1 | 2 | 4 | 1 | 3 | 6 | 3 | 3 | 2 | 2 | 1 | 2 | 4 | 5 | 1 | 3 | 0 | 0 |
| *6–12 years* | 109 | 108 | 100 | 89 | 96 | 112 | 108 | 96 | 98 | 113 | 115 | 114 | 117 | 122 | 132 | 122 | 92 | 101 | 101 | 82 | 42 | 31 |
| *13–17 years* | 79 | 87 | 88 | 84 | 88 | 77 | 75 | 83 | 93 | 100 | 118 | 116 | 110 | 109 | 109 | 102 | 78 | 93 | 90 | 84 | 53 | 47 |
| **GXR⁵** |  |  |  |  |  |  |  |  |  |  |  |  |  |  |  |  |  |  |  |  |  |  |
| *Total* | 556 | 764 | 863 | 892 | 1015 | 1165 | 1237 | 1322 | 1433 | 1512 | 1590 | 1644 | 1596 | 1674 | 1805 | 1784 | 1535 | 1543 | 1529 | 1432 | 730 | 602 |
| *Boys* | 457 | 635 | 714 | 732 | 834 | 958 | 1014 | 1094 | 1168 | 1221 | 1288 | 1339 | 1288 | 1352 | 1436 | 1412 | 1212 | 1208 | 1209 | 1143 | 583 | 482 |
| *0–5 years* | 2 | 2 | 5 | 5 | 7 | 4 | 4 | 5 | 2 | 10 | 10 | 9 | 3 | 5 | 6 | 2 | 6 | 6 | 3 | 3 | 2 | 0 |
| *6–12 years* | 354 | 495 | 530 | 538 | 629 | 731 | 764 | 816 | 858 | 875 | 921 | 955 | 920 | 961 | 990 | 960 | 794 | 787 | 792 | 754 | 387 | 311 |
| *13–17 years* | 101 | 138 | 179 | 189 | 198 | 223 | 246 | 273 | 308 | 336 | 357 | 375 | 365 | 386 | 440 | 450 | 412 | 415 | 414 | 386 | 194 | 171 |
| *Girls* | 99 | 129 | 149 | 160 | 181 | 207 | 223 | 228 | 265 | 291 | 302 | 305 | 308 | 322 | 369 | 372 | 323 | 335 | 320 | 289 | 147 | 120 |
| *0–5 years* | 2 | 1 | 2 | 0 | 0 | 0 | 0 | 0 | 2 | 3 | 4 | 2 | 3 | 2 | 4 | 2 | 3 | 2 | 1 | 1 | 0 | 0 |
| *6–12 years* | 73 | 98 | 110 | 121 | 137 | 154 | 163 | 157 | 178 | 195 | 195 | 202 | 191 | 201 | 234 | 240 | 208 | 211 | 204 | 191 | 101 | 78 |
| *13–17 years* | 24 | 30 | 37 | 39 | 44 | 53 | 60 | 71 | 85 | 93 | 103 | 101 | 114 | 119 | 131 | 130 | 112 | 122 | 115 | 97 | 46 | 42 |

¹Periods represent three-month quarters: Period 1 (April–June), Period 2 (July–September), Period 3 (October–December), and Period 4 (January–March of the following year), following the Japanese fiscal year.

²OROS-MPH: Osmotic controlled-release oral delivery system methylphenidate, ³LDX: Lisdexamfetamine, ⁴ATX: Atomoxetine, ⁵GXR: Guanfacine

**Table S2.** *Trends in the prescription of ADHD medications for new patients*

| *Year* |  | 2018 | |  |  | 2019 | |  |  | 2020 | |  |  | 2021 | |  |  | 2022 | |  | 2023 | |
| --- | --- | --- | --- | --- | --- | --- | --- | --- | --- | --- | --- | --- | --- | --- | --- | --- | --- | --- | --- | --- | --- | --- |
| *Period¹* | 1 | 2 | 3 | 4 | 1 | 2 | 3 | 4 | 1 | 2 | 3 | 4 | 1 | 2 | 3 | 4 | 1 | 2 | 3 | 4 | 1 | 2 |
| **OROS-MPH²** |  |  |  |  |  |  |  |  |  |  |  |  |  |  |  |  |  |  |  |  |  |  |
| *Total* | 103 | 117 | 139 | 102 | 128 | 120 | 100 | 32 | 59 | 76 | 99 | 93 | 77 | 88 | 111 | 78 | 66 | 76 | 71 | 69 | 37 | 19 |
| *Boys* | 78 | 95 | 113 | 81 | 97 | 90 | 86 | 27 | 44 | 60 | 74 | 72 | 63 | 68 | 90 | 54 | 42 | 56 | 52 | 51 | 27 | 15 |
| *0–5 years* | 0 | 2 | 1 | 1 | 0 | 0 | 0 | 0 | 0 | 0 | 0 | 0 | 0 | 1 | 0 | 0 | 0 | 0 | 0 | 0 | 0 | 0 |
| *6–12 years* | 64 | 73 | 87 | 57 | 81 | 72 | 67 | 22 | 35 | 48 | 54 | 46 | 48 | 56 | 72 | 40 | 28 | 48 | 42 | 38 | 23 | 10 |
| *13–17 years* | 14 | 20 | 25 | 23 | 16 | 18 | 19 | 5 | 9 | 12 | 20 | 26 | 15 | 11 | 18 | 14 | 14 | 8 | 10 | 13 | 4 | 5 |
| *Girls* | 25 | 22 | 26 | 21 | 31 | 30 | 14 | 5 | 15 | 16 | 25 | 21 | 14 | 20 | 21 | 24 | 24 | 20 | 19 | 18 | 10 | 4 |
| *0–5 years* | 0 | 0 | 0 | 0 | 0 | 0 | 0 | 0 | 0 | 0 | 0 | 0 | 0 | 1 | 0 | 0 | 0 | 0 | 0 | 0 | 0 | 0 |
| *6–12 years* | 16 | 15 | 15 | 10 | 16 | 16 | 8 | 1 | 8 | 10 | 18 | 7 | 7 | 7 | 11 | 12 | 12 | 9 | 10 | 12 | 5 | 2 |
| *13–17 years* | 9 | 7 | 11 | 11 | 15 | 14 | 6 | 4 | 7 | 6 | 7 | 14 | 7 | 12 | 10 | 12 | 12 | 11 | 9 | 6 | 5 | 2 |
| **LDX³** |  |  |  |  |  |  |  |  |  |  |  |  |  |  |  |  |  |  |  |  |  |  |
| *Total* | 0 | 0 | 0 | 0 | 0 | 0 | 0 | 0 | 0 | 1 | 0 | 0 | 1 | 0 | 2 | 0 | 2 | 1 | 1 | 0 | 0 | 1 |
| *Boys* | 0 | 0 | 0 | 0 | 0 | 0 | 0 | 0 | 0 | 0 | 0 | 0 | 1 | 0 | 1 | 0 | 2 | 0 | 1 | 0 | 0 | 1 |
| *0–5 years* | 0 | 0 | 0 | 0 | 0 | 0 | 0 | 0 | 0 | 0 | 0 | 0 | 0 | 0 | 0 | 0 | 0 | 0 | 0 | 0 | 0 | 0 |
| *6–12 years* | 0 | 0 | 0 | 0 | 0 | 0 | 0 | 0 | 0 | 0 | 0 | 0 | 0 | 0 | 0 | 0 | 1 | 0 | 1 | 0 | 0 | 0 |
| *13–17 years* | 0 | 0 | 0 | 0 | 0 | 0 | 0 | 0 | 0 | 0 | 0 | 0 | 1 | 0 | 1 | 0 | 1 | 0 | 0 | 0 | 0 | 1 |
| *Girls* | 0 | 0 | 0 | 0 | 0 | 0 | 0 | 0 | 0 | 1 | 0 | 0 | 0 | 0 | 1 | 0 | 0 | 1 | 0 | 0 | 0 | 0 |
| *0–5 years* | 0 | 0 | 0 | 0 | 0 | 0 | 0 | 0 | 0 | 0 | 0 | 0 | 0 | 0 | 0 | 0 | 0 | 0 | 0 | 0 | 0 | 0 |
| *6–12 years* | 0 | 0 | 0 | 0 | 0 | 0 | 0 | 0 | 0 | 0 | 0 | 0 | 0 | 0 | 0 | 0 | 0 | 0 | 0 | 0 | 0 | 0 |
| *13–17 years* | 0 | 0 | 0 | 0 | 0 | 0 | 0 | 0 | 0 | 1 | 0 | 0 | 0 | 0 | 1 | 0 | 0 | 1 | 0 | 0 | 0 | 0 |
| **ATX⁴** |  |  |  |  |  |  |  |  |  |  |  |  |  |  |  |  |  |  |  |  |  |  |
| *Total* | 45 | 55 | 50 | 55 | 54 | 65 | 77 | 56 | 55 | 65 | 75 | 71 | 64 | 78 | 79 | 78 | 49 | 70 | 40 | 51 | 27 | 20 |
| *Boys* | 34 | 37 | 33 | 40 | 43 | 42 | 55 | 40 | 36 | 42 | 50 | 41 | 37 | 48 | 52 | 54 | 34 | 46 | 22 | 35 | 17 | 12 |
| *0–5 years* | 0 | 3 | 3 | 1 | 0 | 4 | 3 | 1 | 2 | 2 | 7 | 2 | 1 | 2 | 1 | 1 | 1 | 1 | 0 | 0 | 1 | 1 |
| *6–12 years* | 27 | 20 | 21 | 23 | 30 | 25 | 35 | 27 | 25 | 32 | 32 | 29 | 26 | 35 | 38 | 35 | 25 | 29 | 16 | 25 | 8 | 7 |
| *13–17 years* | 7 | 14 | 9 | 16 | 13 | 13 | 17 | 12 | 9 | 8 | 11 | 10 | 10 | 11 | 13 | 18 | 8 | 16 | 6 | 10 | 8 | 4 |
| *Girls* | 11 | 18 | 17 | 15 | 11 | 23 | 22 | 16 | 19 | 23 | 25 | 30 | 27 | 30 | 27 | 24 | 15 | 24 | 18 | 16 | 10 | 8 |
| *0–5 years* | 0 | 0 | 1 | 0 | 0 | 1 | 0 | 0 | 1 | 3 | 0 | 1 | 0 | 0 | 0 | 0 | 1 | 0 | 0 | 1 | 0 | 0 |
| *6–12 years* | 4 | 7 | 8 | 9 | 4 | 16 | 10 | 7 | 7 | 11 | 11 | 13 | 11 | 14 | 16 | 10 | 9 | 13 | 8 | 6 | 5 | 3 |
| *13–17 years* | 7 | 11 | 8 | 6 | 7 | 6 | 12 | 9 | 11 | 9 | 14 | 16 | 16 | 16 | 11 | 14 | 5 | 11 | 10 | 9 | 5 | 5 |
| **GXR⁵** |  |  |  |  |  |  |  |  |  |  |  |  |  |  |  |  |  |  |  |  |  |  |
| *Total* | 59 | 68 | 76 | 63 | 70 | 93 | 84 | 109 | 71 | 103 | 105 | 118 | 120 | 129 | 152 | 111 | 83 | 93 | 92 | 74 | 45 | 31 |
| *Boys* | 42 | 54 | 59 | 46 | 57 | 74 | 57 | 84 | 48 | 79 | 74 | 87 | 88 | 99 | 104 | 81 | 57 | 65 | 65 | 52 | 33 | 22 |
| *0–5 years* | 1 | 0 | 1 | 0 | 0 | 0 | 0 | 0 | 0 | 6 | 1 | 0 | 0 | 1 | 2 | 0 | 0 | 1 | 0 | 0 | 0 | 0 |
| *6–12 years* | 32 | 42 | 47 | 29 | 50 | 63 | 47 | 70 | 40 | 57 | 59 | 73 | 75 | 88 | 87 | 59 | 41 | 50 | 52 | 43 | 25 | 16 |
| *13–17 years* | 9 | 12 | 11 | 17 | 7 | 11 | 10 | 14 | 8 | 16 | 14 | 14 | 13 | 10 | 15 | 22 | 16 | 14 | 13 | 9 | 8 | 6 |
| *Girls* | 17 | 14 | 17 | 17 | 13 | 19 | 27 | 25 | 23 | 24 | 31 | 31 | 32 | 30 | 48 | 30 | 26 | 28 | 27 | 22 | 12 | 9 |
| *0–5 years* | 1 | 0 | 0 | 0 | 0 | 0 | 0 | 0 | 1 | 0 | 1 | 0 | 1 | 0 | 0 | 0 | 0 | 0 | 0 | 0 | 0 | 0 |
| *6–12 years* | 14 | 12 | 14 | 10 | 9 | 13 | 19 | 13 | 13 | 16 | 21 | 18 | 23 | 20 | 35 | 19 | 16 | 19 | 18 | 14 | 8 | 5 |
| *13–17 years* | 2 | 2 | 3 | 7 | 4 | 6 | 8 | 12 | 9 | 8 | 9 | 13 | 8 | 10 | 13 | 11 | 10 | 9 | 9 | 8 | 4 | 4 |
| **Combination therapy** |  |  |  |  |  |  |  |  |  |  |  |  |  |  |  |  |  |  |  |  |  |  |
| *Total* | 8 | 7 | 7 | 6 | 5 | 14 | 8 | 4 | 8 | 7 | 6 | 3 | 12 | 3 | 5 | 6 | 4 | 10 | 8 | 4 | 1 | 4 |
| *Boys* | 7 | 5 | 7 | 4 | 4 | 12 | 7 | 2 | 6 | 4 | 3 | 3 | 10 | 3 | 4 | 5 | 4 | 7 | 8 | 2 | 1 | 4 |
| *0–5 years* | 0 | 0 | 0 | 0 | 0 | 0 | 0 | 0 | 0 | 0 | 0 | 0 | 0 | 0 | 0 | 0 | 0 | 0 | 0 | 0 | 0 | 0 |
| *6–12 years* | 2 | 4 | 5 | 2 | 2 | 10 | 3 | 0 | 3 | 4 | 3 | 3 | 5 | 2 | 4 | 5 | 2 | 7 | 6 | 1 | 1 | 2 |
| *13–17 years* | 5 | 1 | 2 | 2 | 2 | 2 | 4 | 2 | 3 | 0 | 0 | 0 | 5 | 1 | 0 | 0 | 2 | 0 | 2 | 1 | 0 | 2 |
| *Girls* | 1 | 2 | 0 | 2 | 1 | 2 | 1 | 2 | 2 | 3 | 3 | 0 | 2 | 0 | 1 | 1 | 0 | 3 | 0 | 2 | 0 | 0 |
| *0–5 years* | 0 | 0 | 0 | 0 | 0 | 0 | 0 | 0 | 0 | 0 | 0 | 0 | 0 | 0 | 0 | 0 | 0 | 0 | 0 | 0 | 0 | 0 |
| *6–12 years* | 0 | 2 | 0 | 1 | 1 | 2 | 0 | 0 | 0 | 1 | 2 | 0 | 1 | 0 | 1 | 1 | 0 | 2 | 0 | 0 | 0 | 0 |
| *13–17 years* | 1 | 0 | 0 | 1 | 0 | 0 | 1 | 2 | 2 | 2 | 1 | 0 | 1 | 0 | 0 | 0 | 0 | 1 | 0 | 2 | 0 | 0 |

¹Periods represent three-month quarters: Period 1 (April–June), Period 2 (July–September), Period 3 (October–December), and Period 4 (January–March of the following year), following the Japanese fiscal year.

²OROS-MPH: Osmotic controlled-release oral delivery system methylphenidate, ³LDX: Lisdexamfetamine, ⁴ATX: Atomoxetine, ⁵GXR: Guanfacine

**Table S3.** *Trends in prescription patterns of ADHD medications: monotherapy and combination therapy*

| *Year* |  | 2018 | |  |  | 2019 | |  |  | 2020 | |  |  | 2021 | |  |  | 2022 | |  | 2023 | |
| --- | --- | --- | --- | --- | --- | --- | --- | --- | --- | --- | --- | --- | --- | --- | --- | --- | --- | --- | --- | --- | --- | --- |
| *Period¹* | 1 | 2 | 3 | 4 | 1 | 2 | 3 | 4 | 1 | 2 | 3 | 4 | 1 | 2 | 3 | 4 | 1 | 2 | 3 | 4 | 1 | 2 |
| **Total** | 2874 | 3013 | 3128 | 3096 | 3304 | 3448 | 3525 | 3457 | 3524 | 3737 | 3889 | 3893 | 3849 | 3927 | 4069 | 4014 | 3328 | 3323 | 3313 | 3059 | 1542 | 1297 |
| **Total for monotherapy** | 2366 | 2439 | 2527 | 2490 | 2655 | 2744 | 2822 | 2736 | 2783 | 2957 | 3080 | 3117 | 3086 | 3174 | 3264 | 3204 | 2647 | 2608 | 2610 | 2389 | 1214 | 1033 |
| *OROS-MPH²* | 1398 | 1376 | 1434 | 1391 | 1451 | 1456 | 1480 | 1355 | 1267 | 1351 | 1399 | 1399 | 1411 | 1390 | 1380 | 1365 | 1111 | 1064 | 1078 | 953 | 476 | 416 |
| *LDX³* | 0 | 0 | 0 | 0 | 0 | 0 | 0 | 0 | 4 | 10 | 17 | 22 | 26 | 31 | 34 | 35 | 33 | 36 | 41 | 45 | 23 | 30 |
| *ATX⁴* | 712 | 680 | 642 | 625 | 654 | 638 | 630 | 609 | 671 | 691 | 722 | 685 | 680 | 694 | 718 | 699 | 548 | 578 | 553 | 523 | 263 | 211 |
| *GXR⁵* | 256 | 383 | 451 | 474 | 550 | 650 | 712 | 772 | 841 | 905 | 942 | 1011 | 969 | 1059 | 1132 | 1105 | 955 | 930 | 938 | 868 | 452 | 376 |
| **Total for combination therapy** | 508 | 574 | 601 | 606 | 649 | 704 | 703 | 721 | 741 | 780 | 809 | 776 | 763 | 753 | 805 | 810 | 681 | 715 | 703 | 670 | 328 | 264 |
| *OROS-MPH*  *＋LDX* | 0 | 0 | 0 | 0 | 0 | 0 | 0 | 2 | 4 | 4 | 8 | 6 | 8 | 8 | 11 | 11 | 7 | 14 | 17 | 20 | 9 | 3 |
| *OROS-MPH*  *+ATX* | 208 | 193 | 189 | 188 | 184 | 189 | 178 | 168 | 145 | 166 | 149 | 133 | 125 | 121 | 114 | 111 | 84 | 76 | 84 | 73 | 34 | 31 |
| *OROS-MPH*  *+GXR* | 231 | 286 | 324 | 336 | 384 | 418 | 435 | 431 | 464 | 474 | 502 | 482 | 480 | 468 | 521 | 516 | 443 | 447 | 426 | 407 | 203 | 153 |
| *LDX+ATX* | 0 | 0 | 0 | 0 | 0 | 0 | 0 | 1 | 0 | 1 | 3 | 3 | 3 | 6 | 5 | 7 | 8 | 11 | 9 | 12 | 4 | 3 |
| *LDX+GXR* | 0 | 0 | 0 | 0 | 0 | 0 | 0 | 0 | 3 | 6 | 11 | 13 | 21 | 22 | 28 | 33 | 28 | 36 | 35 | 42 | 17 | 24 |
| *ATX+GXR* | 47 | 72 | 63 | 62 | 60 | 73 | 70 | 96 | 99 | 98 | 102 | 110 | 101 | 102 | 100 | 108 | 90 | 101 | 101 | 93 | 48 | 36 |
| *Triple or more* | 22 | 23 | 25 | 20 | 21 | 24 | 20 | 23 | 26 | 31 | 34 | 29 | 25 | 26 | 26 | 24 | 21 | 30 | 31 | 23 | 13 | 14 |
| **monotherapy proportion (%)⁶** | 82.3 | 80.9 | 80.8 | 80.4 | 80.4 | 79.6 | 80.1 | 79.1 | 79.0 | 79.1 | 79.2 | 80.1 | 80.2 | 80.8 | 80.2 | 79.8 | 79.5 | 78.5 | 78.8 | 78.1 | 78.7 | 79.6 |
| Psychiatry | 85.6 | 84.9 | 81.8 | 85.3 | 84.2 | 77.9 | 80.6 | 83.4 | 83.6 | 87.4 | 88.7 | 84.4 | 81.3 | 84.4 | 83.2 | 81.0 | 79.4 | 77.8 | 84.0 | 82.4 | 82.8 | 79.6 |
| Nonpsychiatry | 82.2 | 80.8 | 80.9 | 80.2 | 80.3 | 79.9 | 80.1 | 78.9 | 79.0 | 78.8 | 78.8 | 79.7 | 80.0 | 80.5 | 80.0 | 79.8 | 79.6 | 78.5 | 78.5 | 77.9 | 78.4 | 79.7 |

¹Periods represent three-month quarters: Period 1 (April–June), Period 2 (July–September), Period 3 (October–December), and Period 4 (January–March of the following year), following the Japanese fiscal year.

²OROS-MPH: Osmotic controlled-release oral delivery system methylphenidate, ³LDX: Lisdexamfetamine, ⁴ATX: Atomoxetine, ⁵GXR: Guanfacine, ⁶Monotherapy proportion (%): Total for monotherapy/Total
